# Lubricant-infused directly engraved nano-microstructures for mechanically durable endoscope lens with anti-biofouling and anti-fogging properties

**DOI:** 10.1038/s41598-020-74517-8

**Published:** 2020-10-15

**Authors:** Yeontaek Lee, Yong-Woo Chung, Jaeho Park, Kijun Park, Youngmin Seo, Seung-No Hong, Seung Hoon Lee, Hojeong Jeon, Jungmok Seo

**Affiliations:** 1 School of Electrical and Electronic Engineering, Yonsei University, 50 Yonsei-ro, Seodaemun-gu, Seoul, 03722 Republic of Korea; 2 Center for Biomaterials, Biomedical Research Institute, Korea Institute of Science and Technology, 5, Hwarang-ro 14-gil, Seongbuk-gu, Seoul, 02792 Republic of Korea; 3 Division of Bio-Medical Science and Technology, KIST School, Korea University of Science and Technology, 5, Hwarang-ro 14-gil, Seongbuk-gu, Seoul, 02792 Republic of Korea; 4 Department of Otorhinolaryngology-Head and Neck Surgery, Boramae Medical Center, Seoul National University College of Medicine, 25 Shindaebang 2-dong, Dongjak-gu, Seoul, 07061 Republic of Korea; 5 Department of Otorhinolaryngology-Head and Neck Surgery, Korea University Ansan Hospital, Korea University College of Medicine, 123, Jeokgeum-ro, Danwon-gu, Ansan, Gyeonggi-do 15355 Republic of Korea; 6 Graduate Institute of Biomedical Engineering, Chang Gung University, No. 259, Wenhua 1st Rd., Guishan Dist., Taoyuan, 33302 Taiwan; 7 Department of Research and Development, Lynk Solutec Inc., 33, Ewhayeodae 3-gil, Seodaemun-gu, Seoul, Republic of Korea

**Keywords:** Medical research, Materials science

## Abstract

While a clear operating field during endoscopy is essential for accurate diagnosis and effective surgery, fogging or biofouling of the lens can cause loss of visibility during these procedures. Conventional cleaning methods such as the use of an irrigation unit, anti-fogging surfactant, or particle-based porous coatings infused with lubricants have been used but proven insufficient to prevent loss of visibility. Herein, a mechanically robust anti-fogging and anti-biofouling endoscope lens was developed by forming a lubricant-infused directly engraved nano-/micro-structured surface (LIDENS) on the lens. This structure was directly engraved onto the lens via line-by-line ablation with a femtosecond laser. This directly engraved nano/microstructure provides LIDENS lenses with superior mechanical robustness compared to lenses with conventional particle-based coatings, enabling the maintenance of clear visibility throughout typical procedures. The LIDENS lens was chemically modified with a fluorinated self-assembled monolayer (F-SAM) followed by infusion of medical-grade perfluorocarbon lubricants. This provides the lens with high transparency (> 70%) along with superior and long-lasting repellency towards various liquids. This excellent liquid repellency was also shown to be maintained during blood dipping, spraying, and droplet condensation experiments. We believe that endoscopic lenses with the LIDENS offer excellent benefits to endoscopic surgery by securing clear visibility for stable operation.

## Introduction

Over the past decade, robotic or laparoscopic surgery has been widely adopted in preference to open surgery due to patient benefits such as reduced risk of infection, faster recovery, and short hospitalization^1^. In addition, endoscopy is used both for diagnostic and therapeutic purposes as the inserted endoscope can visualize, collect biopsy, or remove soft tissue lesions from inside the body. Although a clear operating field of view is highly important in these applications, visibility can be lost because the endoscope lens is prone to biofouling and fogging due to adsorption of body fluids and high humidity. Conventional methods for cleaning the lens include mechanically rubbing the lens against adjacent tissue or introducing additional channels for suction and irrigation^2^. Alternatively, a clear field of view is ensured by retracting the endoscope from the body, wiping it clean, and then re-inserting it^3–5^. However, the aforementioned methods raise concerns regarding possible tissue damage due to rubbing, inconvenience due to the increased size of the endoscope, and the risk of bacterial infection. In view of these issues, it is necessary to develop a novel endoscope lens that can maintain both a clear operating field of view and excellent anti-biofouling properties for accurate diagnosis and effective surgical procedures.

To address some of these problems, surface modification has been used to engineer the material design and chemical properties of the lens. For example, Yu et al.^6^ used a plasma etching method to form microstructures on glass, generating a superhydrophilic surface that could effectively prevent fogging and maintain visibility in a humid environment by inducing film-like condensation of water^7,8^. However, the surface was vulnerable to biofouling and the lens tended to become opaque due to the adhesion of non-transparent and viscous body fluids. To prevent biofouling, a superhydrophobic surface that mimics the lotus leaf has attracted considerable attention^9,10^. For instance, Jokinen et al.^11^ developed superhydrophobic coatings with nano-/micro-structures for medical applications. The surface allowed the formation of a thin layer of air pockets between the surface and any contacting liquid, thus repelling blood and small quantities of body fluids. However, in spite of its excellent anti-biofouling property, the superhydrophobic surface has been shown to be vulnerable to fogging as nucleated droplets adhere to the nano-/micro-structure^12^. Unfortunately, hydrophilicity and hydrophobicity are incompatible properties and neither property can solve both fogging and biofouling at the same time^13^.

With a view to overcoming the aforementioned limitations, lubricant-infused porous surfaces inspired by the surface of the *Nepenthes* pitcher plant have recently received significant attention^14^. These surfaces have been shown to not only repel biologically relevant adsorbates, including body fluids, proteins, and bacteria, but also to prevent fogging by providing a drastically low nucleation density^12,15^.

Sunny et al.^16^ reported an anti-fouling material fabricated via the layer-by-layer (LbL) deposition of charged particles to form hierarchical micro porous structures with infiltrated lubricants. However, because the fabrication process involved the introduction of additional material onto the substrate, the particle-based porous structure was not durable under mechanical stimulus because the particles could be torn apart. Additional mechanical limitations arising from the introduction of additional materials onto the surface include weak interactions between different materials and difficulty in forming a well-ordered and uniform structure, both of which are critical for the stability and reliability of the coating. Hence, attempts have been made to overcome the mechanical weakness of additive coatings by developing the porous structure directly on the material via the femtosecond (fs) laser-engraving technique^15,17–19^. This approach has advantages due to its availability, relatively low cost, high processing speed, and wide variety of patterning effects. Moreover, in contrast to nanosecond or continuous laser techniques, the very high energy density of the fs laser light (due to the extremely short pulse width of 10^–15^–10^–12^ s) allows it to be absorbed even on transparent objects with minimum heat damage to the sample. In addition, since nano/microstructure fabrication via fs laser surface pattering involves the direct engraving of the substrate, this enables the production of structures with highly ordered and finely adjustable topographies along with higher mechanical durability than that achieved by the additive method. Nevertheless, there have not been any previous studies on the use of fs lasers to fabricate a nano/microstructure endoscope lens with superior anti-biofouling, and anti-fogging properties.

In this paper, the lubricant-infused directly engraved nano-/micro-structured surface (LIDENS) is presented as a simple but effective approach to the fabrication of an endoscope lens with enhanced mechanical durability along with anti-fogging and anti-biofouling properties. The superior mechanical robustness of the LIDENS compared to that obtained by the additive method is demonstrated under cyclic linear abrasion and tape-peeling tests, wherein the initial surface roughness and anti-biofouling characteristics are maintained even after 30 cycles. In addition, the formation of the optimal surface structure and lubricant layer is found to provide an average optical transparency to visible light of > 70%. Moreover, the anti-biofouling properties of LIDENS are confirmed in the presence of various liquids and proteins e.g. blood and simulated body fluid (SBF), with a sliding angle of ≤ 10º. Finally, since the loss of visibility during endoscopy is expected to be caused by fogging (~ 80 °C, 100% relative humidity; RH) as well as biofouling, the performance of the LIDENS-integrated endoscope system is demonstrated in simulated physiological environments via blood spraying at an angle of 45º to the lens and via repeated dipping of the endoscope in blood. The low nucleation density and dynamic coalescence of the LIDENS are shown to enable the removal of droplets under gravity, thus preventing fogging and possible biofouling over 80% of the visible area.

## Results and discussion

**Figure 1:**
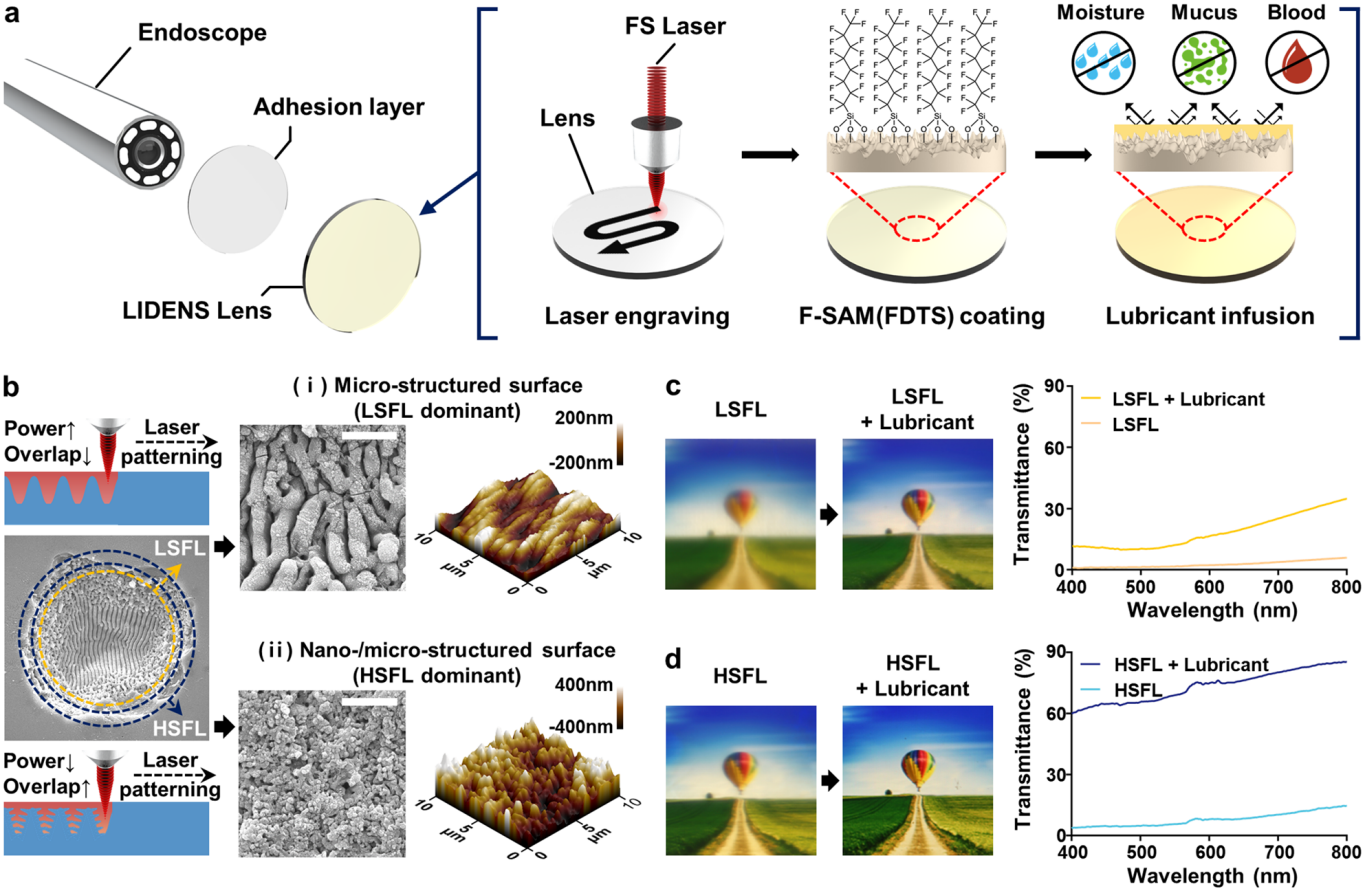
**(a)** A schematic illustration of the anti-biofouling and anti-fogging endoscope lens and its fabrication process; **(b)** SEM and AFM images of the directly engraved (i) microstructure surface and (ii) nano/microstructure surfaces according to fs laser power and overlap degree (scale bar, 2 μm); **(c,d)** Transmittance before and after injection of lubricant on **(c)** LSFL and **(d)** HSFL structured bk6 glass surface.

Conventionally, nano-/micro-structures have been made by depositing additives such as nanoparticles or by lithographically patterning using photo-sensitive polymers^20,21^. However, these methods often involve complex fabrication processes and toxic chemicals. In addition, the deposited nanoparticles and polymers are not mechanically durable and can be easily detached from the surface, thus hindering practical applications. To overcome these limitations, a fs laser was employed in the present work to directly engraved nano-/micro-structures onto the endoscope lens without degrading the mechanical durability. The directly engraved nano-/micro-structured surface (DENS) was then coated with a fluorinated self-assembled monolayer (F-SAM), followed by infiltration of the lubricants to generate the LIDENS. The long fluorocarbon chains in the F-SAM reduce the surface energy of DENS, thus increasing the chemical affinity between the surface and medical-grade lubricants. This allows easy infiltration of the lubricants into the nano/microstructures, thus providing LIDENS with extreme anti-fouling and anti-fogging properties. These properties, along with the fabrication procedure of the endoscope lens, are shown schematically in Fig. 1a. In order to maximize the transparency after lubricant infusion, four different perfluorocarbon-based lubricants, including perfluorodecalin (PFD), GPL 100, GPL 101, and GPL 103 were compared using UV–visible spectroscopy. The refractive index of GPL 103 was found to be similar to that of the endoscope lens (Figure S1, Supporting Information), thus providing the optimum transmittance. Hence, GPL 103 was used for all further experiments.

The fs laser engraving process produces laser-induced periodic surface structure (LIPSS) on the lens surfaces via line-by-line laser scanning. Depending on the laser power, two different types of LIPSS can be formed. These are referred to as the high-spatial frequency LIPSS (HSFL) and the low-spatial frequency LIPSS (LSFL). Previous studies have demonstrated that the LSFL displays microscale roughness, whereas the HSFL displays nanoscale roughness^22^. Here, the optimum power of laser irradiation was determined by observing the differences in the surface topographical patterns according to the laser power and number of laser pulses (Figure S2, Supporting Information). This demonstrated that the LSFL was primarily formed at the center of the engraved area, while the HSFL was formed at the rim area. The relative area of each structure could be modulated by controlling the power, with a high area ratio of HSFL being obtained at low power, and the area ratio of LSFL increasing as the laser power was increased.

For the engraving of surface structures over a large area via line-by-line scanning, various combinations of overlap region and laser power of were investigated. Examination of the resulting surfaces by scanning electron microscopy (SEM) and atomic force microscopy (AFM) (Fig. 1b) indicated that (i) a higher pulse energy of 87.84 J/cm^2^ combined with a smaller overlap (line distance = 10 µm) resulted in more LSFL-dominant microstructures while, conversely, (ii) a lower pulse energy of 2.44 J/cm^2^ combined with a higher overlap (line distance = 5 µm) resulted in more HSFL-dominant nano/microstructures. Such structural differences in LSFL-dominant and HSFL-dominant surfaces have led to changes in the optical and wettability properties^23–25^. The difference in transparency of the HSFL and LSFL surfaces was observed by comparing the visibility of an image of a hot-air balloon (which is used for eye-tests) and by comparing the transmittance levels via UV–visible spectroscopy (Fig. 1c,d) on bk6 glass. Prior to infusion of the lubricant into the surface, the transmittance of the HSFL surface was slightly (~ 9%) higher than that of the LSFL surface.

Recent studies suggest that the surface roughness plays an important role in determining the optical properties because the scattering behavior changes according to the level of surface roughness, thus affecting the transparency of the glass substrate^26–28^. This is in line with the present results that the LSFL surface has a lower transmittance than that of the HSFL, since the LSFL surface has the larger microstructures in which more scattering can occur. Furthermore, the drastic increases in the transparency of both the LSFL and HSFL observed after infiltration of the lubricant can be explained by a significant reduction in the light scattering due to the smoothing out of the rough surface structure by the lubricant, as suggested by previous studies^29^. However, the degree of the improvement obtained was found to vary depending on the surface topography. Thus, while the LSFL (in which the micro-scale structure predominated) could only transmit ~ 28% of the visible light even after lubricant injection (Fig. 1c), the HSFL (in which the nano-scale structure predominated) displayed an eightfold increase in transmittance after lubricant injection and was able to transmit 84% or more of the visible light (Fig. 1d). These results suggest that the HSFL with smaller nano-/micro-structures improve the transmissivity and the effective lubricant retention compared to LSFL in which its microstructure might have not held lubricant fully. Hence, further experiments were conducted on the LIDENS with the HSFL.

**Figure 2:**
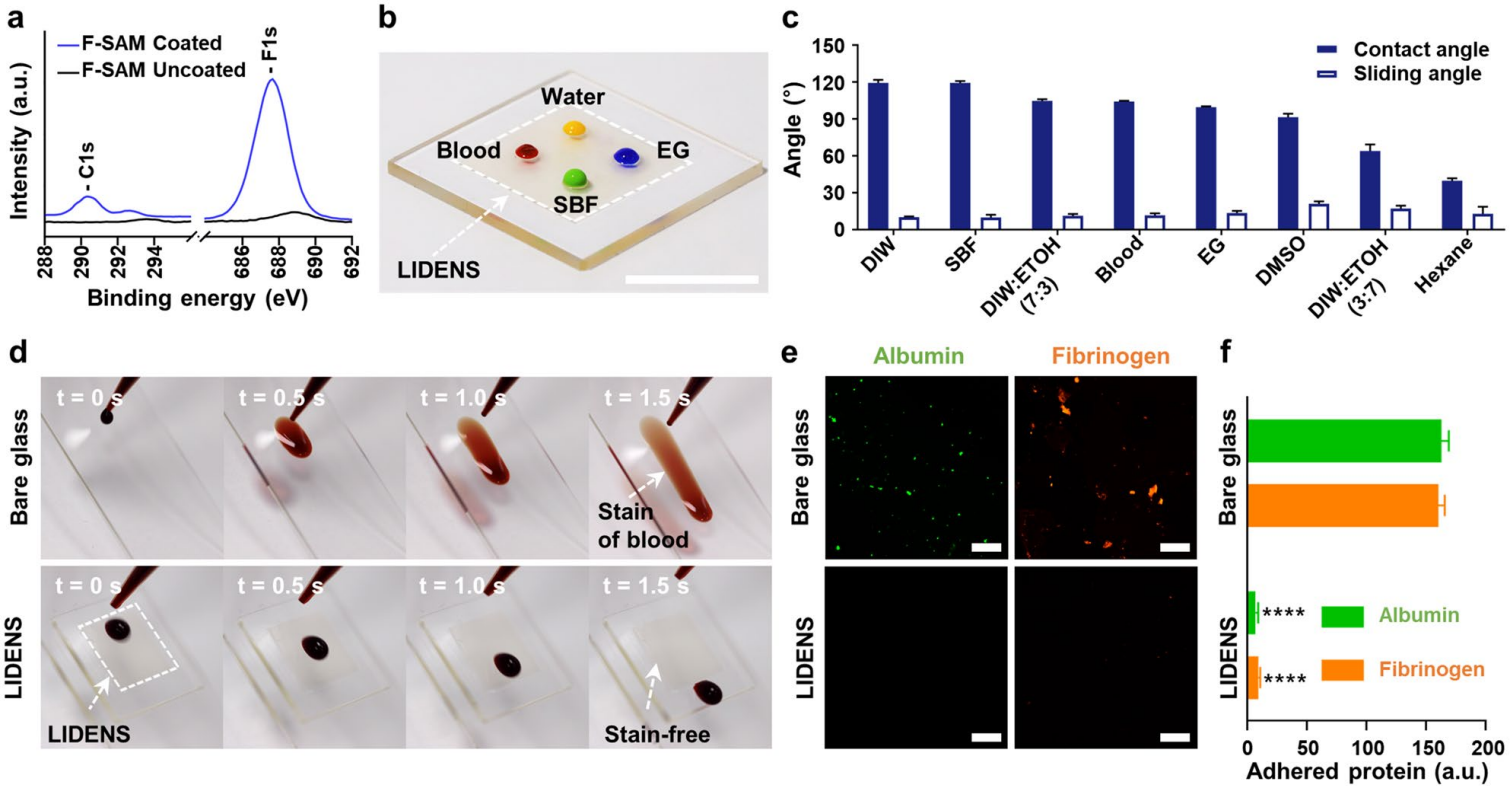
**(a)** XPS spectra of fs-laser engraved samples with and without F-SAM formation on the surface; **(b)** photographic images of various liquids on the LIDENS (scale bar: 1 cm); **(c)** CA and SA of various liquid droplets on the LIDENS; **(d)** sequential photographs of blood droplets (volume ~ 50 μL) on bare glass and the LIDENS samples at a tilt angle of 45°; **(e)** confocal microscopy images of protein (albumin and fibrinogen) adsorptions on bare and LIDENS samples (scale bar: 100 μm).

To identify any changes in the chemical characteristics due to surface functionalization, the chemical compositions of DENS with and without the F-SAM coating were characterized by X-ray photoelectron spectroscopy (XPS). Thus, the XPS spectra in Fig. 2a reveal a significant increase in the F1s peak at ~ 688 eV and a moderate increase in the C1s peak at ~ 290 eV for the F-SAM coated sample compared to the uncoated sample. In addition, Table S1 (Supporting Information) indicates the increased atomic ratios of fluorine and carbon over oxygen and silicon in the F-SAM-coated DENS, thus confirming the successful formation of the F-SAM coating.

Due to the combination of low surface energy from the F-SAM and the DENS, the coated surface displayed excellent liquid-repellency properties similar to those previously reported for the lotus leaf structures^30^. The surface wettability of the DENS with and without the F-SAM coating were investigated by comparing the respective liquid contact angles (CA) (Figure S3, Supporting Information). Thus, it was confirmed that the unmodified DENS was completely wetted (CA ≤ 5°) by a range of liquids including deionized (DI) water and blood, with surface tensions of > 58 mN/m^31^, as well as dimethyl sulfoxide (DMSO) and ethylene glycol (EG), with surface tensions of < 43 mN/m. This superhydrophilicity can be attributed to the intrinsic hydrophilicity of the glass being enhanced by the nano-/micro-structure of the DENS^32,33^. By contrast, the DENS coated with F-SAM displayed extremely high CAs (> 160°) for both water and blood, yet still showed pinning behavior for DMSO and EG with low surface tension. Although the F-SAM coating might increase the CA by reducing the surface energy and the area of contact between the surface and the liquids, the structure of DENS induces a capillary force that enables wetting with low surface energy liquids. Therefore, since wetting cannot be prevented in superhydrophobic surfaces that mimic the lotus leaf, this may eventually lead to loss of visibility when applied to an endoscope. However, this limitation can be addressed by infusing a lubricant with extremely low surface energy into the F-SAM-coated DENS to produce LIDENS. Thus, the representative optical image of various liquid droplets on the LIDENS shown in Fig. 2b indicates the extreme liquid repellency of LIDENS due to the change from surface-liquid interface to lubricant-liquid interface as the lubricant was infused. The CAs and sliding angles (SAs) for various liquid droplets on the LIDENS were then analyzed. As indicated in Fig. 2c, the LIDENS consistently displayed a very low SA of ≤ 15° in spite of any reduction in CA arising from the different surface tensions of the various liquids, even including 70 vol% ethanol solution and hexane which have very low surface tensions of < 30 mN/m.

In addition, the adhesion of droplets of blood was examined on both the LIDENS and the bare glass surface in order to confirm the excellent anti-biofouling properties of LIDENS. As shown by the sequential images in Fig. 2d, a blood droplet (~ 10 µL) was dropped onto each surface at an angle of 45° in order to observe the surface adhesion while sliding. The blood droplet was observed to slide for a relatively long time (> 3 s) on the bare glass, thus leaving blood stains (i.e. biofouling) on the surface. By contrast, the blood droplet was observed to slide for less than 1.5 s on the LIDENS without leaving any stains or residues, thus demonstrating the excellent anti-biofouling property of LIDENS.

The adsorption of plasma protein by the bare glass and the LIDENS was tested by immersing each sample for 24 h in blood mixed with either albumin or fibrinogen, followed by fluorescence microscope imaging. As indicated in Fig. 2e,f, significant amounts of albumin and fibrinogen were adsorbed onto the bare glass surface, whereas negligible adsorption was observed on the LIDENS.

**Figure 3:**
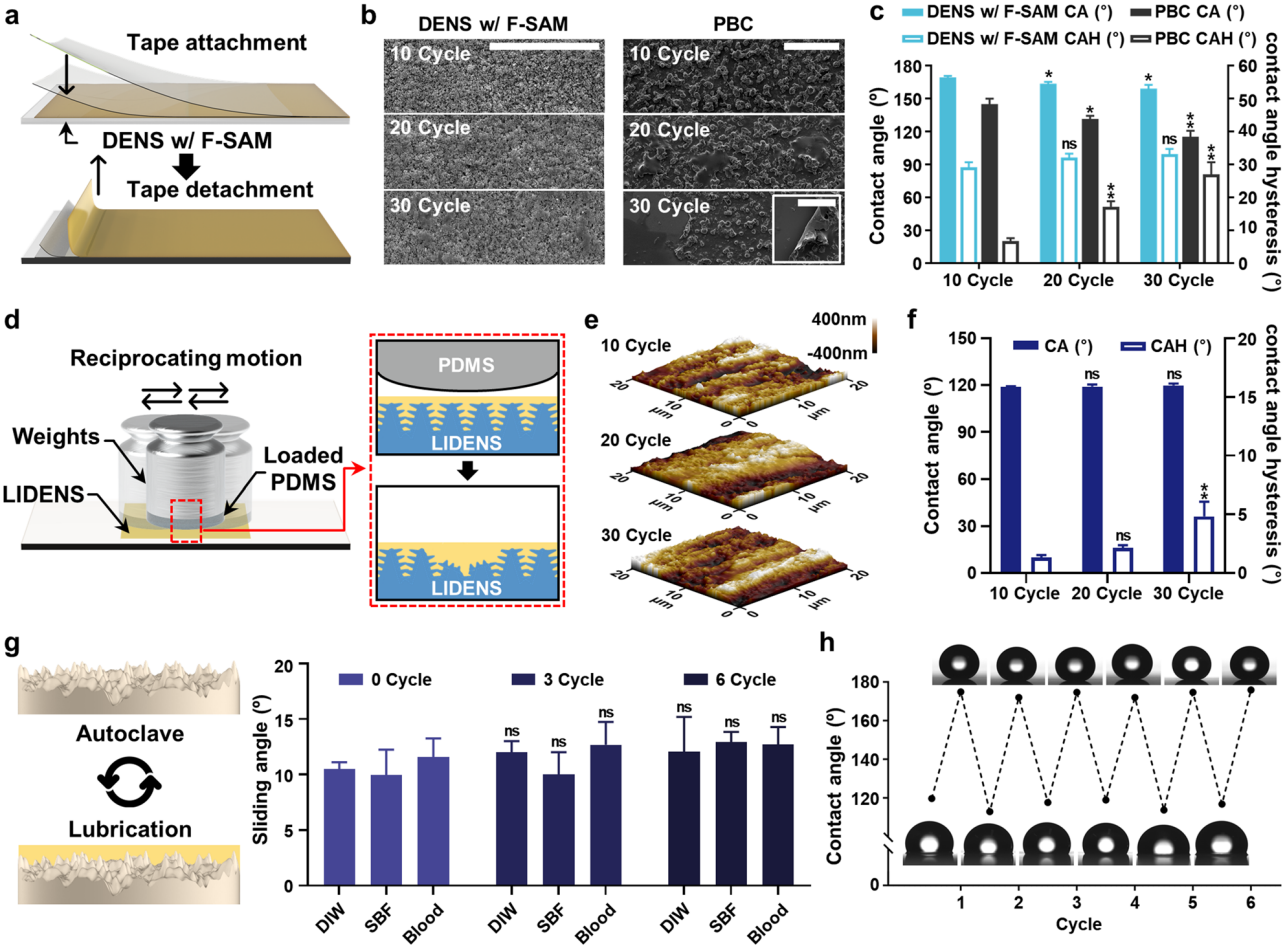
**(a)** A schematic illustration of the tape-peeling tests for DENS w/F-SAM, performed using a sticky tape; **(b)** SEM images showing the changes in the morphology of DENS with F-SAM and the commercially available particle-based coating (PBC) after 10, 20, 30 tape-peel test cycles (scale bars: 20 and 200 μm for DENS w/F-SAM and PBC, respectively); **(c)** changes in the CA and CAH according to repeated tape-peeling tests; **(d)** a schematic illustration of the linear abrasion tests for LIDENS; **(e)** representative AFM images showing the morphologies of LIDENS after 10, 20, 30 cycles of linear abrasion tests; **(f)** changes in the CA and CAH according to repeated linear abrasion tests; **(g)** measured SA for LIDENS during repeated sterile autoclave cycles and lubrication process; **(h)** reversible CA during the repeated autoclave and lubrication process.

The above-mentioned series of in vitro experiments confirmed the excellent anti-biofouling property of LIDENS, with a low SA regardless of the type of liquid and its surface tension. The anti-biofouling properties of LIDENS were also demonstrated with respect to body fluids such as blood and plasma protein solutions. However, since the endoscope will be exposed to mechanical stimuli such as abrasion and compression due to contact with the surrounding tissues during endoscopic surgery, the mechanical stability and durability must also be considered when designing the lens coating. Therefore, the mechanical durability of LIDENS with directly engraved surface topography was tested against various mechanical stimuli. The cyclic tape-peeling tests applied to the DENS with the F-SAM coating, and to a commercially available particle-based coating (PBC)^34^, are shown schematically in Fig. 3a. After 30 cycles of tape peeling, the SEM images in Fig. 3b indicate no significant topological changes in the DENS coated with F-SAM, whereas the PBC became detached and eventually lost its liquid repellency.

To further investigate the effects of the tape-peeling tests on the functions of the DENS coated with F-SAM, the CA and contact angle hysteresis (CAH) were analyzed as indicators of liquid repellency (Fig. 3c). The CA measures the ability of the liquid to wet the material surface, whereas the CAH measures the surface heterogeneity. DENS w/F-SAM sample showed a low CAH (~ 7°) before the tape-peeling experiment. Right after the tape-peeling experiment, CAH of DENS was observed as ~ 30° following the slight increase in CAH with repeated tape-peeling action (< 10°). There are two possible reasons for the CAH rise right after the tape-peeling test. First, pieces of adhesive layer of tape, which are relatively less hydrophobic, were adsorbed on the DENS (Figure S4). Second, a slight damage of protruding nano-/micro-structure while using 1 kg steel roller to ensure full contact between the surfaces and adhesive tape might have resulted in the exposure of the hydrophilic bare glass to the surface. The described local defects caused droplet pinning on the surface, which soon led to CAH rise. However, these defects were very local and did not affect the overall surface chemical affinity with the lubricant or since the damaged area is covered by the infused lubricant, the partial damage to the nano/microstructure is likely to be negligible in practical use. By contrast, a significant decrease in the CA (> 30º), along with an increase in the CAH (> 45º), was observed for the PBC as the particles were peeled off and the particle adhesive layer was torn apart. The sharp increase in CAH for the PBC suggests an increase in surface non-uniformity due to coating detachment. This suggests that the technique described herein for coating DENS with F-SAM provides a superior nano/microstructure with greater mechanical resistance to structural degradation than that obtained by conventional additive-based methods.

In addition, a linear abrasion test was performed in order to confirm the mechanical durability of LIDENS against the bumping and sweeping often experienced by endoscope lenses when coming into contact with adjacent tissues during surgical procedures. As shown schematically in Fig. 3d, the abrasion environment was simulated by attaching 0.5 kg weights onto an abradant to ensure complete contact between the LIDENS and the abradant. After repeated linear abrasion tests of 10, 20 and 30 reciprocating motion cycles, changes in the roughness and topography of the nano/microstructure were characterized by AFM, as shown in Fig. 3e. In addition, Figure S5 (Supporting Information) indicates that as the number of reciprocating motion cycles increased, both the R_a_ and R_q_ values decreased slightly while a high surface roughness (R_a_ > 80 nm, R_q_ > 99 nm) was maintained. This suggests that these mechanical stimuli applied to the endoscope lens have minimal effect on the topography. In addition, the effect of the linear abrasion test upon the anti-biofouling and anti-fogging properties was examined by measuring the associated changes in the CA and CAH. The results presented in Fig. 3f demonstrate a slight rise in CA (< 2º) and CAH (< 4º) after repeated abrasive motion, which might be attributed to slight loss of the lubricant. It suggests that the anti-biofouling and anti-fogging properties of LIDENS are not affected by abrasion because the lubricant covers and spontaneously replenishes the damaged area via surface energy-driven capillary action^35^.

In addition to the above-mentioned requirements, coatings for medical applications must be durable against essential sterilization steps such as washing by sonication or sterilization by high-temperature, high-pressure autoclave. For the lubricated surface coating, any loss of lubricant during sterilization might be considered as a factor degrading the performance of the surface. Therefore, LIDENS was exposed to six repeated cycles of sterilization including autoclave at 121 °C for 15 min, with each cycle being followed by re-lubrication, and any change in the SA were measured using DI water, SBF, and blood. The results presented in Fig. 3g indicate that the evaporation of the lubricant was accelerated by the high-temperature, high-pressure environment, which lead to a slight decrease in the SA. After the additional lubrication step, however, the SA was recovered and found to be below 15º (Fig. 3g). In addition, the stability of the F-SAM coating during repeated autoclaving was confirmed by sonicating the LIDENS in washing solution to completely remove the lubricant and then measuring the CA after each step (Fig. 3h). After complete removal of the lubricant, the LIDENS showed same surface characteristics as the DENS coated with F-SAM (CA > 160°). When additional lubricant was subsequently applied to the surface, the performance of the LIDENS was seen to be fully recovered and showed no degradation when exposed to further sterilization steps. In addition, long-term stability of LIDENS was investigated (Figure S6, Supporting Information). LIDENS maintained its sliding angle even after 28 days of mild shaking (60 rpm) in PBS. This is due to the high chemical affinity between DENS w/ F-SAM and perfluorocarbon-based lubricant enabling formation of a stable lubricant layer. The results of the above-mentioned tests suggest that LIDENS is mechanically stable and capable of maintaining its anti-biofouling properties even after repeated cycles of stimuli such as tape peeling, linear abrasion, and sterilization. By contrast, degradation in the performance of the conventional additive method (PBC) was observed due to loss of the surface structure or to particles being torn apart.

**Figure 4:**
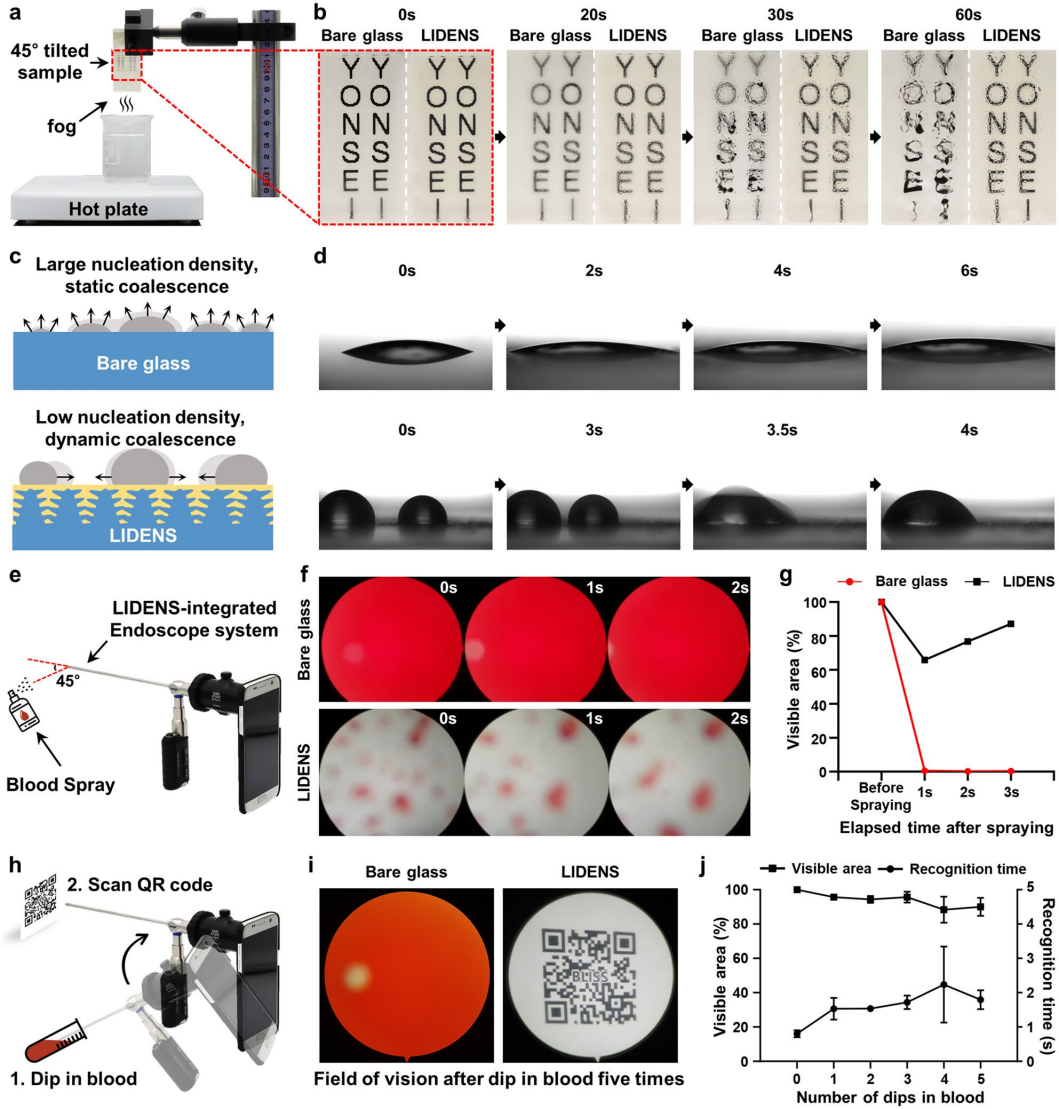
**(a)** A schematic illustration of the set-up for anti-fogging tests; **(b)** sequential photographic images of the bare glass (left) and LIDENS (right) after being held at ~ 3 cm over steaming water (~ 80 °C, 100% RH) for 60 s; **(c)** a schematic illustration of the two factors influencing droplet nucleation depending on the surface characteristics (i.e. nucleation density and droplet coalescence); **(d)** sequential photographs of droplet coalescence on the bare glass surface (top) and LIDENS (bottom); **(e)** a schematic of the small droplet adsorption test set-up for the LIDENS-integrated endoscope system using blood spray; **(f)** sequential images showing the field of vision for the untreated glass (top) and the LIDENS (bottom); **(g)** visible area percentage after blood spraying on the bare endoscope and the LIDENS-integrated endoscope system. **(h)** A schematic illustration of the blood dipping test set-up; **(i)** field of vision after the blood dipping test for the untreated glass (left) and LIDENS (right); **(j)** visible area percentage after the blood dipping tests.

To further investigate the ability of LIDENS to retain a clear field of vision during endoscopic surgical procedures, anti-fogging and anti-blood adhesion tests were performed, as shown in Fig. 4. First, the anti-fogging performance of LIDENS was tested by means of a dropwise condensation experiment. For ease of comparison, the LIDENS was formed on one-half of a square glass sample and a piece of printed paper was attached to the opposite side of the coated surface. As shown in Fig. 4a, the sample was positioned above steaming water (~ 80 °C, 100% relative humidity (RH)), and the temperature of the hotplate was adjusted to maintain the continuous condensation of vapor as water droplets. The sequential images in Fig. 4b clearly demonstrate that the vapor condensate slid off the LIDENS, thus allowing the surface to maintain its operating field of view without any visual distortion, whereas adsorbed droplets formed on the bare glass surface to significantly compromise its field of vision. As shown schematically in Fig. 4c, these findings can be explained from two perspectives, namely: (i) nucleation density and (ii) droplet coalescence. First, the LIDENS showed a lower nucleation density compared to that of the bare glass surface. A recent study on droplet condensation suggested that the number of nucleating droplets is dramatically lower on hydrophobic samples compared to hydrophilic samples^36^. Since the LIDENS contains infused lubricant with extremely low surface energy, the resulting hydrophobicity would be expected to lead to less droplet nucleation and, hence, clearer visibility. Second, LIDENS showed high droplet mobility which resulted in dynamic coalescence of droplets, as indicated by the lower set of sequential photographic images in Fig. 4d and Movie S1 (Supporting Information). Given that the pinning force is proportional to the CAH^13^, the low CAH of LIDENS would drastically reduce the pinning force of the droplet, thus enabling high surface mobility and potential coalescence of the droplet with adjacent droplets. This dynamic coalescence of the droplets on the LIDENS resulted in increased droplet size, thus allowing the droplets to be removed as gravity overcome the surface retention force^12^. By contrast, the upper set of sequential photographic images in Fig. 4d demonstrate that the high CAH of the bare glass surface would increase the pinning force of the droplets, thereby reducing their surface mobility by keeping them firmly pinned so that only static coalescence could occur. This indicates that the field of vision of the bare glass is easily reduced and distorted due to the static coalescence of highly pinned droplets, thus requiring wiping to restore visibility, whereas the LIDENS surface maintains its visibility in the humid environment.

Beside fogging due to droplet condensation and coagulation, loss of visibility due to biofouling due to the adherence of body fluids on the lens can cause problems that hinder accurate diagnosis and effective surgical procedures. Thus, the anti-biofouling property is also essential for securing stable endoscopic visibility. Therefore, in vitro test of the anti-biofouling performance of LIDENS was performed by attaching a glass substrate with the LIDENS onto an endoscope and combining this with a smartphone with a specially made case, adapter, and portable light source to illuminate the field of view through an embedded light guide. This LIDENS-integrated endoscope system was used to confirm the anti-biofouling performance by simulating endoscopic surgical situations such as blood dipping and blood spraying. The experimental set-up for the small droplet (≤ 1 µL) adsorption test by spraying blood at the endoscope is shown schematically in Fig. 4e, while the sequential images in Fig. 4f and graph in Fig. 4g indicate the resulting visible area of the endoscopic lens. The top row of images in Fig. 4f indicates that the blood droplets were adsorbed on bare endoscope lens and spread out due to the surface hydrophilicity, thus resulting in immediate and total vision loss. By contrast, the bottom row of images demonstrates the occurrence of dynamic coalescence on the LIDENS, which resulted in the aggregation of small adsorbed blood droplets into larger droplets which then slid off. This, in turn, lead to the gradual recovery of visibility. Recent studies on the mechanism of water droplet movement on solid surfaces and the minimum droplet size required for overcoming any retention force^16,37^ suggest that, since the surface infused with lubricant has a retention force of less than 10 µN, the droplet must weigh more than 10 µN (volume ≥ 1 µL) in order to be coalescent and removed by gravity. Nevertheless, a few small droplets remained adsorbed on the LIDENS even after dynamic coalescence when the gravitational force acting on the droplet was less than the retention force. This can be explained by the complex composition and lower surface tension (58 mN/m) of the blood used in the present experimental work, which may have enhanced the retention force and increased the minimum droplet volume needed for removal by the gravitational force. However, these droplets can be easily removed by a gentle shake or repeated dipping.

As shown schematically in Fig. 4h, the occurrence of severe bleeding during endoscopy was simulated by immersing the endoscope in blood five consecutive times then measuring the time taken to recognize the QR code. An examination of the left-hand image in Fig. 4i and Movie S2 (Supporting Information) indicates that the endoscope with the bare glass was fully covered by blood and lost its field of vision, making it impossible to recognize the QR code after the dipping test. By contrast, the right-hand image indicates that the LIDENS retained sufficient clarity for recognition of the QR code. In addition, the percentage of visible area and the corresponding QR code recognition time were measured for each blood dipping step, and the results for the LIDENS endoscope are presented in Fig. 4j. Here, a partial loss of visibility is evident after the fourth dip, indicating that a blood droplet was retained on the surface. Nevertheless, the visibility was fully recovered as the blood droplet was removed after further dipping. Thus, the excellent anti-biofouling and anti-fogging properties of LIDENS were demonstrated by its ability to retain visibility over 80% of its area.

In conclusion, the exceptional anti-biofouling and anti-fogging properties of LIDENS provide a possible solution for the loss of endoscopic visibility due to the presence of body fluids or a humid environment. By modulating the fluence and overlapping the fs laser pulses, it was possible to form mechanically robust and nano-/micro-structures which are mechanically appropriate for use in medical applications. In addition, subsequent surface functionalization was by F-SAM enhanced the chemical affinity of the substrate to the medical-grade perfluorocarbon-based lubricant, thus facilitating the infusion of the lubricant into the nano/microstructure. This approach was used to construct a LIDENS lens with transparent, anti-biofouling and anti-fogging properties. The excellent anti-biofouling property of LIDENS was confirmed on the micro and macro scale by the extremely low SA demonstrated by using various liquids with different surface tensions, and by the lack of adsorption of blood droplets and plasma protein. In addition, LIDENS showed mechanical robustness under various circumstances including the tape-peeling test, linear abrasion test, and repeated autoclave test, during which its CA, CAH, and surface topography were maintained. This suggests that LIDENS is capable of enduring harsh conditions that may occur in a surgical environment. Finally, LIDENS was applied to a rigid endoscope lens to confirm its clear operating field even in extreme in vitro tests designed to mimic the actual endoscopic environment, including high-humidity, blood spraying, and blood soaking. Hence, LIDENS is a promising coating for a endoscope lens that affords resistance to biofouling and fogging in a surgical environment. Moreover, LIDENS could be applied in industrial endoscopes used for dirt-filled sewer pipelines, boilers, turbines, or chemical plants.

## Methods

### Fabrication of LIDENS on endoscope lens

An fs laser system (s-pulse HP, Amplitude, France) with a pulse duration of 400 fs, a wavelength of 343 nm, and a repetition rate of 1 kHz was employed for laser surface patterning on bk7 glass for endoscope lenses. All samples were laser engraved under ambient air. The laser beam spot size and scan speed were fixed at 110 μm in diameter and 4 mm/s, respectively. In the present study, two laser conditions were used to fabricate nano/microstructures on the glass substrate. With a fixed laser fluence of 87.84 J/cm^2^ and a line-to-line laser beam distance of 10 μm, LSFL were dominant on the surface. However, nano-/micro-structures (HSFL) were formed when the substrate was irradiated with the laser fluence of 2.44 J/cm^2^ and the line-to-line distance was 5 μm. The engraved endoscope lenses were plasma treated (FEMTO SCIENCE, South Korea) under ambient oxygen (100 W, 5 min) and located in a vacuum chamber (0.08 MPa) with a glass vial containing 100 μL of 1H, 1H, 2H, 2H-perfluorooctyltriethoxysilane (FOTS, Sigma-Aldrich, Germany) to form a self-assembled monolayer (SAM). After coating with the FOTS SAM layer, the endoscope lens was cleaned with isopropyl alcohol (IPA) and deionized (DI) water to remove excess FOTS. To form LIDEN, a perfluorocarbon-based lubricant (GPL103, Krytox Performance Lubricants, Du Pont, USA) was applied to the FOTS-coated lenses. The fabricated LIDEN endoscope lenses were tilted at an angle of 45° and left overnight to remove the excess lubricants.

### Characterization

Surface morphologies were examined using a field emission microscopy (SEM; Inspect F50, FEI, USA) and atomic force microscopy (AFM; XE-100, Park Systems, Korea). The optical transmittance of the endoscope lens was characterized using an ultraviolet and visible (UV–vis) spectrophotometer (S-3100, SCINCO, Korea) with the wavelength ranging from 400 to 800 nm. The transmittance spectrum was collected using air as a reference. The chemical compositions of the samples were analyzed using an X-ray photoelectron spectroscopy (XPS) system (PHI 5000 VersaProbe, ULVAC-PHI, Japan) equipped with an Al K-α X-ray source. Fluorescence images were acquired using confocal laser scanning microscopy (LSM 700, Carl Zeiss, Oberkochen, Germany), and were analyzed using the Zeiss ZEN confocal software. Liquid CA, CAH and SA were measured by using a contact angle measurement system equipped with a dynamic image capture camera (Smart Drop, FEMTOBIOMED, Korea). For the liquid CAH and SA measurements, tilted-drop measurement was conducted using 5 μL droplets of various liquids (DI water, horse blood, ethylene glycol, dimethyl sulfoxide, simulated body fluid). The tilted-drop method begins with placing a droplet of each liquid on the samples and gently tilting at 0.5° per second until the droplet rolled off. The angle at which the droplet rolled off is the SA, whereas the CAH is the measured difference between the advanced and receding CA just before the droplet rolls off the surface (n = 5).

### Protein adsorption test

Alexa Fluor 488 conjugated albumin from bovine serum (BSA) (A13100, Invitrogen) and fibrinogen from human plasma (F13191, Invitrogen) were purchased from Invitrogen and used without further purification. To evaluate the anti-adhesion properties of LIDENS endoscope lenses to plasma protein, the bare and LIDENS endoscope lenses were immersed into 5 mL of aqueous protein solutions (1 mg/mL) and incubated at 37° C for 24 h. The samples were then washed with DI water and dried under atmospheric conditions. The protein adsorption on the LIDENS endoscope lens was characterized using a confocal microscope (LSM 700, Carl Zeiss, Oberkochen, Germany), and quantified by the ImageJ/FIJI software program (https://imagej.nih.gov/ij/).

### Linear abrasion test

To confirm the abrasion resistance of the laser engraved structures, a laboratory-scale wear resistance test machine was used. A 10 mm-thick polydimethylsiloxane (PDMS; Sylgard 184, Dow Corning, USA) abrasive was obtained by mixing 200 g of PDMS with a curing agent in a weight ratio of 10:1 and gently pouring into a round polystyrene Petri dish (140 mm in diameter) after degassing in a vacuum chamber. The PDMS substrate was cured in an oven at 80 °C for 1 h, then carefully peeled off and cut into a 3 cm-diameter circle. Then, the PDMS abrasive was attached under a 0.5 kg weight and mounted on an automated XY translational stage to apply frictional stress to the endoscope lenses. The abrasive was directly contacted to the surface of the sample and repeatedly moved back and forth 30 times over a distance of 2 cm.

### Adhesive tape-peeling test

A strong adhesive tape (VHB; 3 M) with an adhesion force of 2600 N/m was attached to the DENS and to the fluorinated silica nanoparticle (F-NP) coated surface^38^ via a steel roller. To ensure full contact between the surfaces and adhesive tape, the tape was pressed twice with a 1 kg steel roller and then peeled off. Here, the tape adhesion and peel-off steps comprised one cycle and a fresh tape was used for each cycle. After each peeling test cycle, the CA and CAH were measured using the characterization method described above.

### Analysis for the stability of the lubricant layer under flow

Stability of lubricant layer in PBS was monitored by measuring sliding angles. LIDENS samples were submerged in 5 mL of PBS for each well of a 6-well plate and gently shaken using orbital shaker at 60 rpm. The sliding angle of each samples were measured with 2 day intervals until day 14 and final measurement was performed on the 28th day.

### Blood fouling test

Laboratory experiments were performed using defibrinated horse blood (Kisanbio, Korea) to simulate the visual degradation caused by blood fouling during endoscopic surgery. A glass substrate with LIDENS was cut into a 4 mm-diameter circle and attached onto the rigid endoscope lens (Telescope, Mega Medical) using PDMS as an adhesion layer. Then, the endoscope and smartphone camera (galaxy S7, Samsung, Korea) were combined with a specially-made phone case, adapter, and portable light source (Smart scope, KARL STORZ, Germany). The blood fouling resistance of the endoscope lenses was examined via blood dipping and spray tests. For the blood dipping test, the end of the endoscope was immersed in the blood and withdrawn for five consecutive times. To test the visibility of the endoscope lenses, a photograph of the printed QR code was taken with the attached camera and the captured image was analyzed by examining the acquired area of view and checking whether the QR code could be recognized. For the spray tests, the time taken for visibility to be recovered sight after the first degradation was determined by spraying blood onto the endoscope from a distance of 20 cm at a tilt angle of 45°.

### Anti-fogging test

To test the anti-fogging property, the LIDENS was formed on one-half of a 2 cm × 2 cm glass sample and a piece of printed paper was attached to the opposite side. The sample was then positioned 5 cm above steaming water (~ 60 °C, 100% RH) at a tilt angle of 45°, and the temperature of the hot plate was adjusted to 80 °C to maintain the continuous condensation of water vapor to form droplets. At the same time, droplet nucleation onto sample, and the resulting level of transparency, was recorded by time-lapse photographic imaging (~ 60 s) with a digital camera (Canon, Japan).

### Statistical analysis

The statistical analysis was performed using the Graphpad Prism software (Graphpad Software Inc., USA). The differences between groups were assessed by the unpaired t-test, ordinary two-way ANOVA, and Tukey’s multiple comparison test, with individual variances computed for each comparison. The levels of significance are indicated in the Figures as follows: *p < 0.05; **p < 0.01; ***p < 0.001; ****p < 0.0001; ns: not significant.

## Supplementary information

Supplementary Information.

Supplementary Video 1.

Supplementary Video 2.
