# Supplementary material for: Lubricant-infused directly engraved nano-microstructures for mechanically durable endoscope lens with anti-biofouling and anti-fogging properties
Source: Sci Rep. 2020 Oct 15;10:17454. doi: 10.1038/s41598-020-74517-8 (PMC7566624; doi:10.1038/s41598-020-74517-8)
Supplement: Supplementary file 1 — Supplementary Information. [file 41598_2020_74517_MOESM1_ESM.docx]

Supporting Information

**Lubricant-infused Directly Engraved Nano-microstructures for Mechanically Durable Endoscope Lens with Anti-biofouling and Anti-fogging Properties**

*Yeontaek Lee ^a,g^, Yong Woo Chung ^b^, Jaeho Park ^b^, Kijun Park ^a^, Youngmin Seo ^a,b^, Seung-No Hong ^d^, Seung Hoon Lee^e^, Hojeong Jeon ^b,c,^*, and Jungmok Seo ^a,f,^**

^a^ School of Electrical and Electronic Engineering, Yonsei University, 50 Yonsei-ro, Seodaemun-gu, Seoul 03722, Republic of Korea

^b^ Center for Biomaterials, Biomedical Research Institute, Korea Institute of Science and Technology, 5, Hwarang-ro 14-gil, Seongbuk-gu, Seoul 02792, Republic of Korea

^c^ Division of Bio-Medical Science and Technology, KIST School, Korea University of Science and Technology, 5, Hwarang-ro 14-gil, Seongbuk-gu, Seoul 02792, Republic of Korea

^d^ Department of Otorhinolaryngology-Head and Neck Surgery, Boramae Medical Center, Seoul National University College of Medicine, 25 Shindaebang 2-dong, Dongjak-gu, Seoul 07061, Republic of Korea

^e^ Department of Otorhinolaryngology-Head and Neck Surgery, Korea University Ansan Hospital, Korea University College of Medicine, 123, Jeokgeum-ro, Danwon-gu, Ansan-city, Gyeonggi-do, 15355, Republic of Korea

^f^ Graduate Institute of Biomedical Engineering, Chang Gung University, No. 259, Wenhua 1st Rd., Guishan Dist., Taoyuan City 33302, Taiwan

^g^ Department of Research & Development, Lynk solutec Inc., 33, Ewhayeodae 3-gil, Seodaemun-gu, Seoul, Republic of Korea

Corresponding Author:

[jeonhj@kist.re.kr](mailto:jeonhj@kist.re.kr) (Dr. Hojeong Jeon),

[jungmok.seo@yonsei.ac.kr](mailto:Jungmok.seo@yonsei.ac.kr) (Prof. Jungmok Seo)

Keywords: Endoscope lens, Anti-biofouling, Anti-fogging, Femtosecond laser, Nano-/micro- structured surface


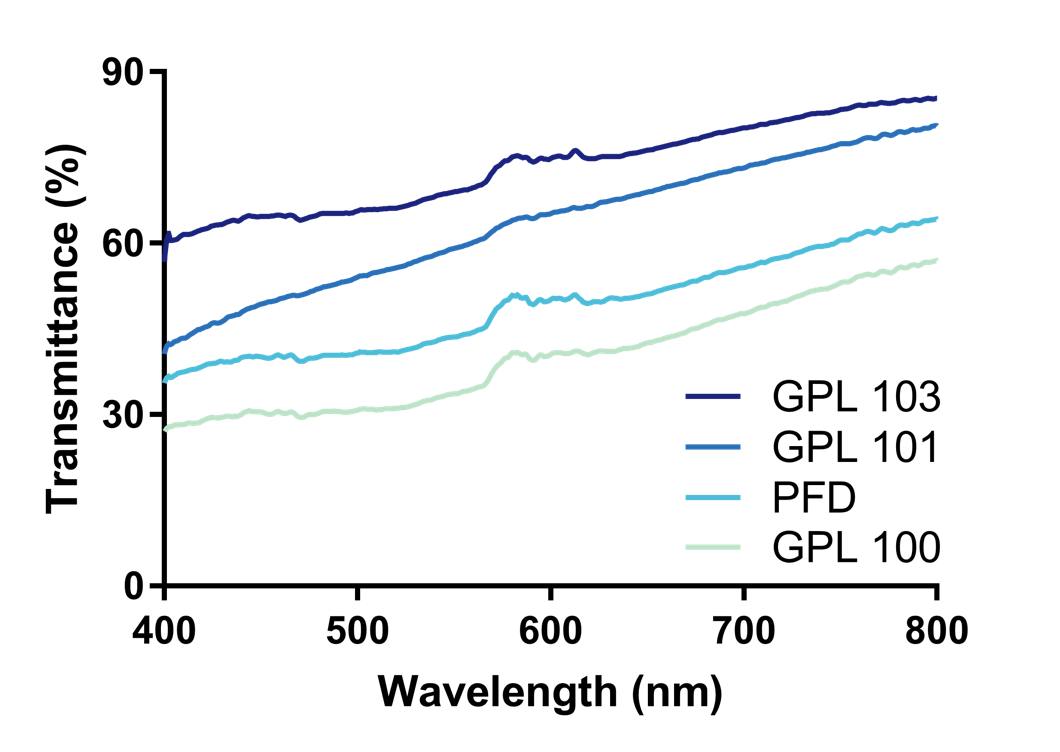


**Figure S1**. Transmittance spectra of LIDENS with various perfluorocarbon-based lubricants.


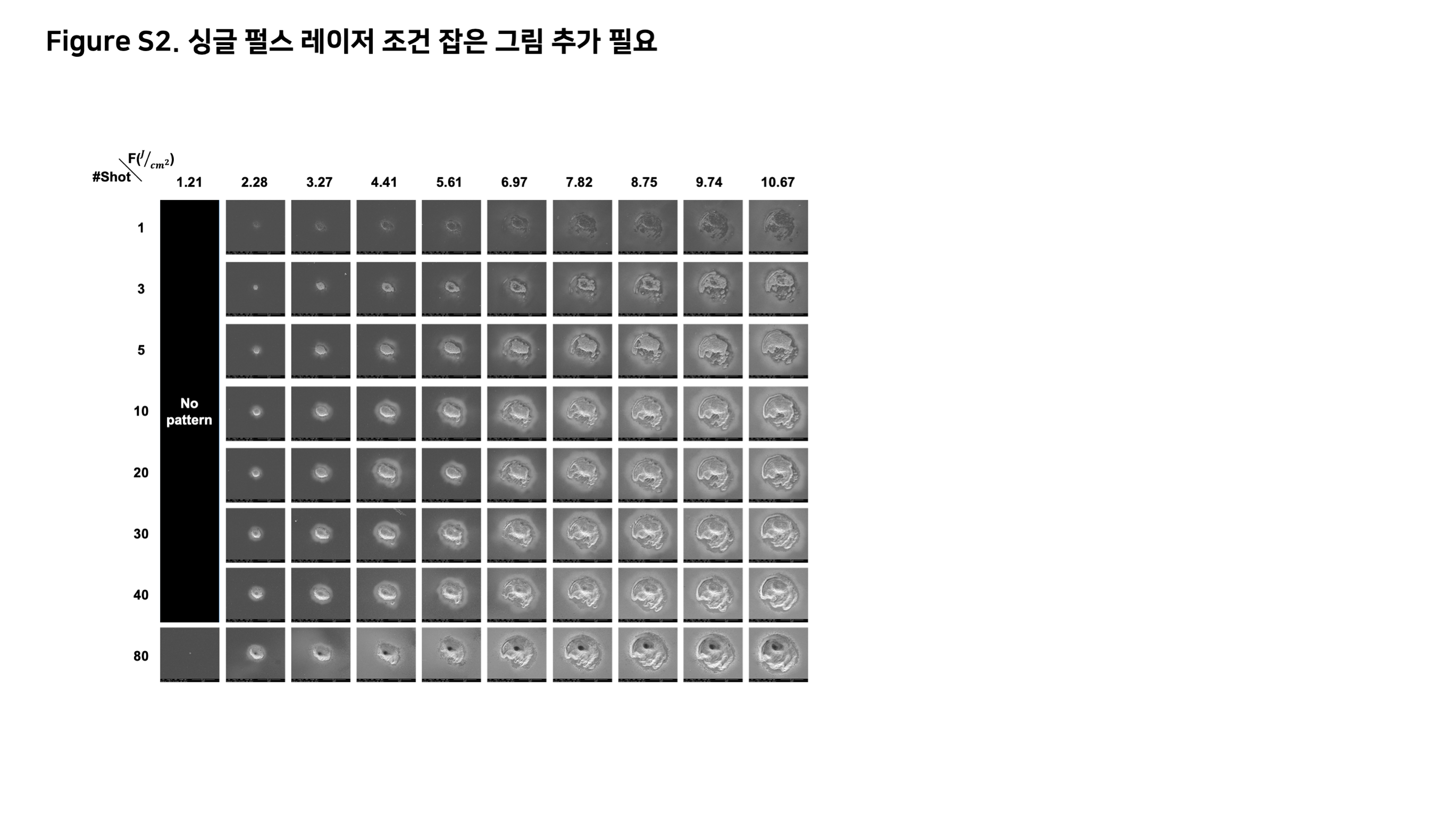


**Figure S2**. Single pulse pattern changes according to the number of shots and power (fluence; J/cm^2^). This is used to determine the optimum laser power for the formation of LSFL or HSFL over a relatively large area.

| Peak | | C 1s | N 1s | O 1s | F 1s | Si 2p | K 2p |
| --- | --- | --- | --- | --- | --- | --- | --- |
| Atomic  Concentration  (%) | DEN | 6.89 | 0.00 | 61.09 | 4.81 | 26.31 | 0.91 |
|  | DEN with F-SAM | 33.10 | 0.00 | 10.00 | 50.54 | 6.36 | 0.00 |

**Table S1**. XPS peaks indicating the atomic concentrations of various components of the untreated DENS and the DENS with F-SAM. Based on the increased C and F ratio, it can be seen that F-SAM is well-formed on the DENS.

*
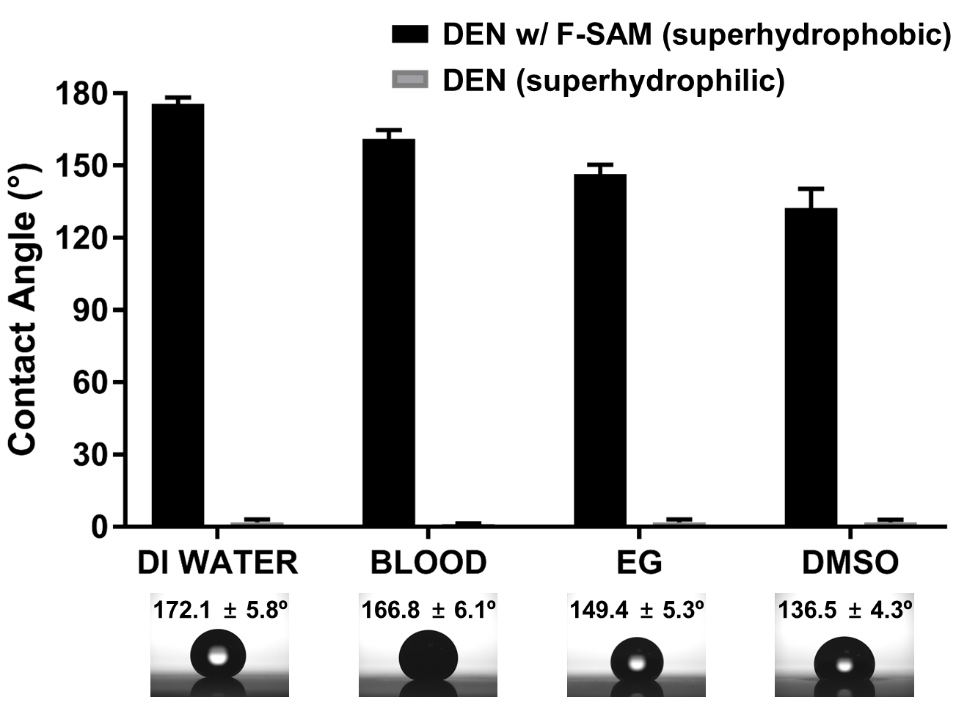
*

**Figure S3**. The CA of deionized (DI) water, blood, ethylene glycol (EG), and dimethyl sulfoxide (DMSO) on DENS and DENS with F-SAM.


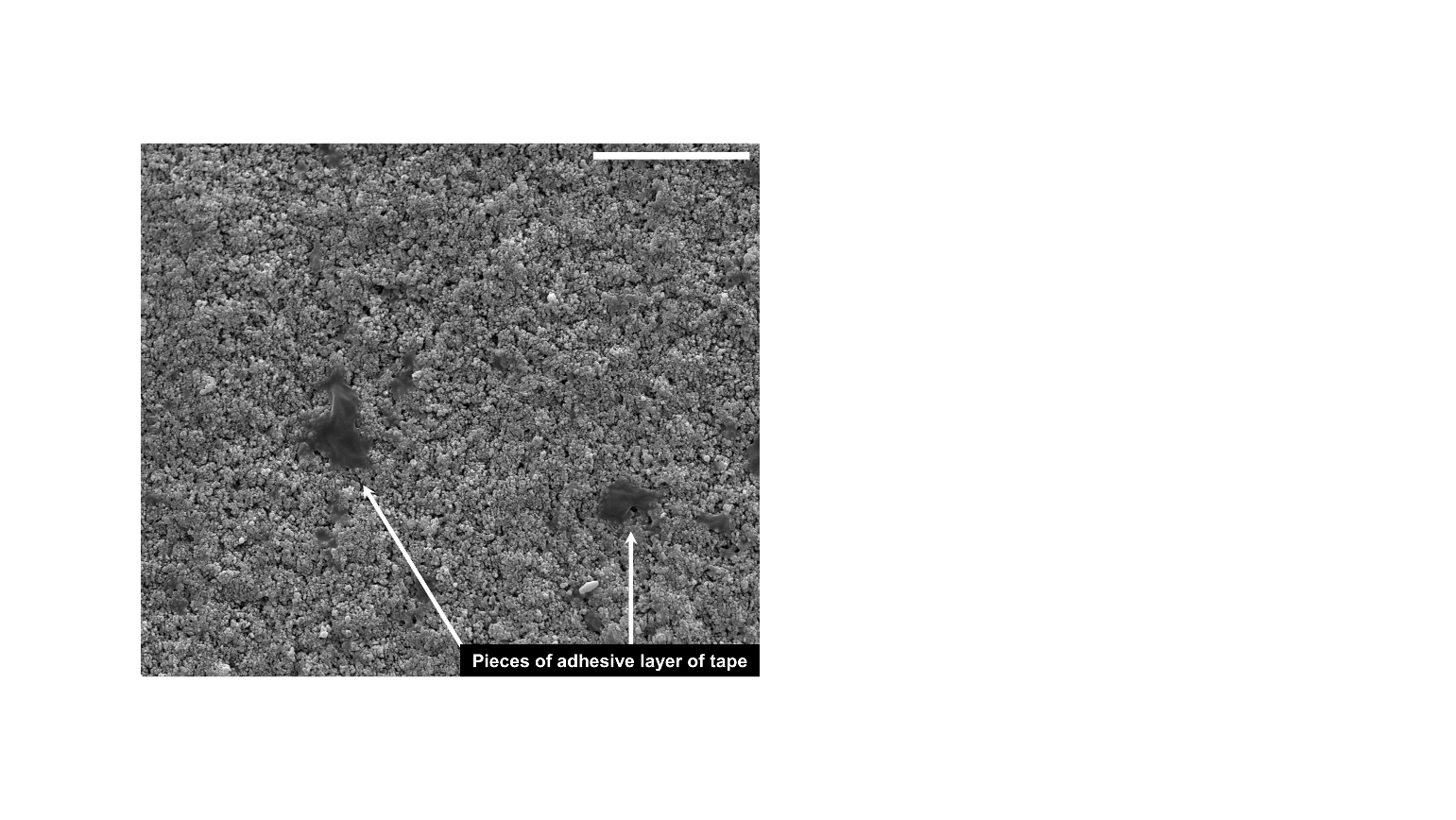


**Figure S4**. SEM image of the surface where the adhesive of the tape is partially absorbed after the tape peeling test. (Scale bar, 10 μm)

*
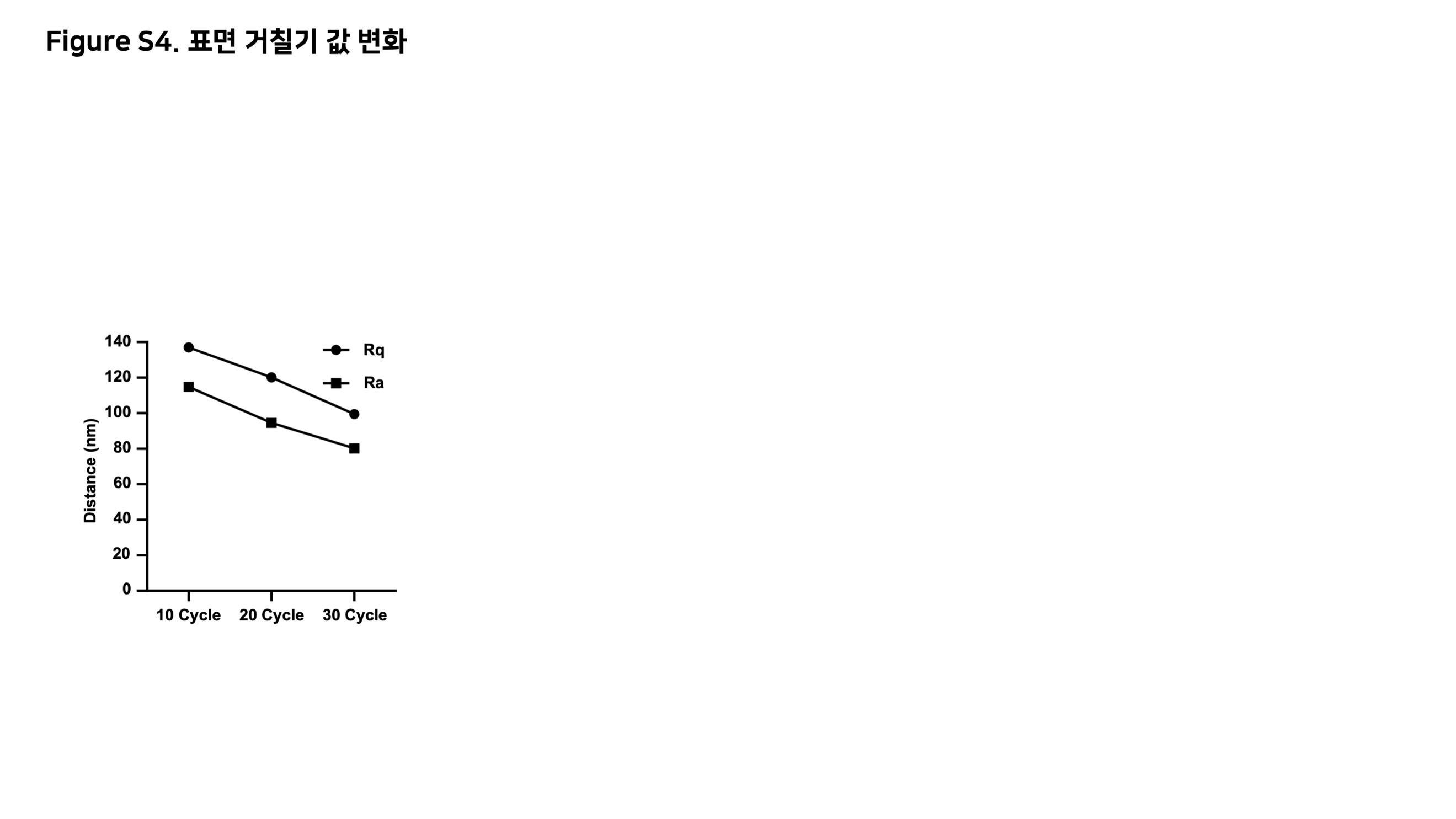
*

**Figure S5**. Surface roughness after 10, 20, 30 cycles of linear abrasion tests.


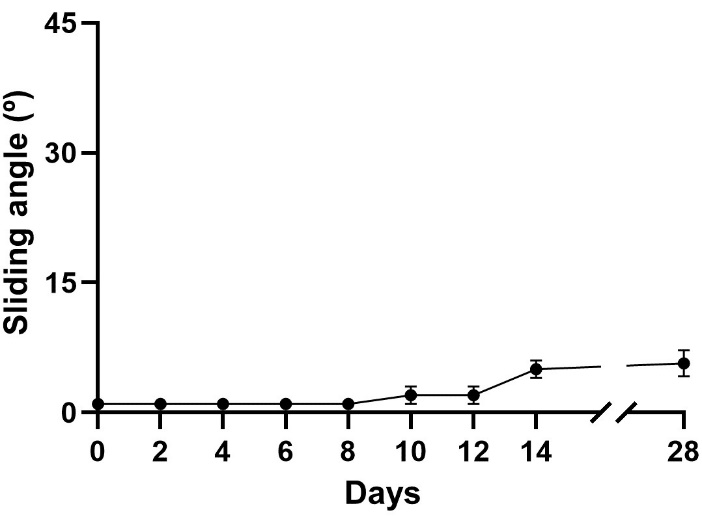


**Figure S6**. Long-term stability analysis of LIDENS samples through sliding angle test.
